# Nitrous Oxide (N_2_O) Emissions by Termites: Does the Feeding Guild Matter?

**DOI:** 10.1371/journal.pone.0144340

**Published:** 2015-12-10

**Authors:** Alain Brauman, Muhammad Zeeshan Majeed, Bruno Buatois, Alain Robert, Anne-Laure Pablo, Edouard Miambi

**Affiliations:** 1 IRD, UMR ECO&SOLS, Campus Supagro, Montpellier, France; 2 IRD, UMR Eco&Sols, LMI LUSES, Land Development Department, Bangkok, Thailand; 3 CNRS, UMR 5175 CEFE, Montpellier, France; 4 IRD, iEES, Bondy, France; 5 Université Paris-Est Créteil, iEES, Créteil, France; The University of Akron, UNITED STATES

## Abstract

In the tropics, termites are major players in the mineralization of organic matter leading to the production of greenhouse gases including nitrous oxide (N_2_O). Termites have a wide trophic diversity and their N-metabolism depends on the feeding guild. This study assessed the extent to which N_2_O emission levels were determined by termite feeding guild and tested the hypothesis that termite species feeding on a diet rich in N emit higher levels of N_2_O than those feeding on a diet low in N. An in-vitro incubation approach was used to determine the levels of N_2_O production in 14 termite species belonging to different feeding guilds, collected from a wide range of biomes. Fungus-growing and soil-feeding termites emit N_2_O. The N_2_O production levels varied considerably, ranging from 13.14 to 117.62 ng N_2_O-N d^-1^ (g dry wt.)^-1^ for soil-feeding species, with *Cubitermes* spp. having the highest production levels, and from 39.61 to 65.61 ng N_2_O-N d^-1^ (g dry wt.)^-1^ for fungus-growing species. Wood-feeding termites were net N_2_O consumers rather than N_2_O producers with a consumption ranging from 16.09 to 45.22 ng N_2_O-N d^-1^ (g dry wt.)^-1^. Incubating live termites together with their mound increased the levels of N_2_O production by between 6 and 13 fold for soil-feeders, with the highest increase in *Capritermes capricornis*, and between 14 and 34 fold for fungus-growers, with the highest increase in *Macrotermes muelleri*. Ammonia-oxidizing (*amoA*-AOB and *amoA*-AOA) and denitrifying (*nirK*, *nirS*, *nosZ*) gene markers were detected in the guts of all termite species studied. No correlation was found between the abundance of these marker genes and the levels of N_2_O production from different feeding guilds. Overall, these results support the hypothesis that N_2_O production rates were higher in termites feeding on substrates with higher N content, such as soil and fungi, compared to those feeding on N-poor wood.

## Introduction

Nitrous oxide (N_2_O) is one of the main greenhouse gases in the atmosphere [1] and contributes to the destruction of the ozone layer [2]. Although global annual emissions of N_2_O have been estimated at 17.7 Tg N [3], the relative contributions from individual sources have not yet been determined.

In tropical terrestrial ecosystems, termites are the most abundant soil macrofauna and have a wide trophic diversity. By virtue of microbial symbionts in their gut, termites play a key role in ecosystem processes such as carbon and nitrogen mineralization [4–6]. Little is known about the extent to which termites contribute to the atmospheric N_2_O budget in tropical ecosystems, although the metabolic activity of termite gut microbiota maintains steep oxygen gradients within the gut lumen [7], which may help to allow nitrification and denitrification processes to occur simultaneously. These two microbial processes are the principal sources of N_2_O emissions, either as a by-product of nitrification or as an intermediate in denitrification [8]. Genes that encode for the enzymes involved in the nitrification process include ammonium monooxygenase (*amo*), hydroxylamine oxidoreductase *(hao)* and nitrite oxidoreductase (*nxr*) whereas those that conduct denitrification include nitrite reductases (*nirK*, *nirS*), nitric oxide reductase (*norB*), and nitrous oxide reductase (*nosZ*). The genes *amo*, *nirK*, *nirS* and *nosZ* are most commonly used as functional markers to assess the nitrification and denitrification processes [9]. Recently, the use of denitrifying gene abundance as an indicator of greenhouse gas emissions from soils was suggested by Morales et al. [10].

Mineralization of soil nitrogen by termites has mainly been investigated for soil-feeding termite species. Unlike wood-feeding termites which thrive on nutrient-poor material containing as little as 0.05% N [11], soil-feeding species are able to feed on nitrogenous soil components (peptides, proteins and amino acids) as shown by feeding experiments [12–14]. High ammonia concentrations have been detected throughout the termite gut with a considerable accumulation in the posterior hindgut [13]. A study using the ^15^N-tracer technique showed that denitrification in soil microcosms was stimulated in the presence of termites [15]. Nitrate ingested by soil-feeding termites was denitrified to N_2_ or reduced to ammonia in the gut [15]. Ngugi and Brune [16] also used the ^15^N-tracer technique to study the fate of nitrate in the gut compartments of *Ophiotermes* spp. and two *Cubitermes* species. Their study was the first report on N_2_O production in living individuals of soil-feeding termite species and in their gut homogenates after soil-feeding termite mounds had been identified as hot spots of N_2_O emissions in the African savannah [17]. However, Majeed et al. [18] recently showed that wood-feeding termites were able to take up atmospheric N_2_O. The fate of nitrogen in termite guts depends on the feeding guild [19]. Therefore, understanding the relationship between feeding behavior and N_2_O emissions is a critical step in estimating the global contribution of termites to the N_2_O emission budget in tropical forests.

This study tested the hypothesis that termite species feeding on a diet rich in N emit higher levels of N_2_O than those feeding on a diet low in N. It set out to assess the extent to which termite feeding behavior affects N_2_O levels and addressed the differences in N_2_O production for termite species belonging to various feeding guilds, collected from a wide range of biomes. It also tested for relationships between the feeding guild and the levels of N_2_O production, the abundance of marker genes for ammonia oxidation (*amoA* and *amoB*) and denitrification (*nirK*, *nirS* and *nosZ*) processes, and mineral N concentration in termite guts. So far as we are aware, this is the first study to use a single dataset to compare N_2_O production rates by termite species belonging to different feeding guilds and to report the presence of ammonia-oxidation and denitrification marker genes in termite guts.

## Materials and Methods

### Ethics Statement

This study did not involve endangered or protected species. The termites used in this study were collected from the termite rearing laboratory at the Institut de Recherche pour le Développement (IRD) in Bondy (France) with the permission of Dr. Corinne Rouland-Lefèvre, IRD research director–Ile de France.

### Termite collection and breeding conditions

Termites can be classified into four broad feeding guilds: wood-feeders, soil-feeders, fungus-growers and grass-feeders. Fifteen termite species from colonies kept in laboratory conditions belonging to different feeding guilds were used (Table 1). Termite colonies were collected from different sites in tropical and subtropical biomes and kept in the laboratory at the Institute of Research for Development (IRD) at Bondy (France) on a 12-h light/dark cycle. They were kept at 27°C ± 2°C and 80% relative humidity with a constant diet of wood, soil or grass depending on their feeding guild. Worker termites were used for all experiments because they are the most numerous caste and are responsible for all the work in the colony.

**Table 1:** Termite species studied along with their colony collection sites.

Feeding Group	Species	Family	Origin	Biome	Collection Site	Geographical repartition
Fungus-growing	*Macrotermes muelleri*	Termitidae	Congo	Rainforest	Dimonika(04° 11′ 51″ S—12° 20′ 09″ E)	Afrotropical, Oriental
	*Pseudacanthotermes militaris*	Termitidae	Gabon	Savanna	Bateke Plateaux(02° 09′ 27″ S—14° 00′ 26″ E)	Afrotropical
	*Pseudacanthotermes spiniger*	Termitidae	Gabon	Rainforest	Franceville(01° 37′ 15″ S—13° 34′ 58″ E)	Afrotropical
Grass-feeding	*Coarctotermes clepsydra*	Termitidae	Madagascar	Savanna	Ambohimanambola(19° 48′ 30″ S—46° 37′ 03″ E)	Malagasy
	*Hodotermes mossambicus*	Hodotermitidae	South Africa	Dry savanna	Pretoria(25° 44′ 13″ S—28° 16′ 50″ E)	Afrotropical
	*Trinervitermes* spp.	Termitidae	Congo	Rainforest	Pointe noire(04° 46′ 43″ S—11° 51′ 49″ E)	Afrotropical, Oriental
Soil-feeding	*Capritermes capricornis*	Termitidae	Madagascar	Savanna	Mananara(16° 29′ 32″ S—49° 42′ 42″ E)	Malagasy
	*Crenetermes albotarsalis*	Termitidae	Congo	Rainforest	Dimonika(04° 07′ 09″ S—12° 21′ 12″ E)	Afrotropical
	*Cubitermes* spp.	Termitidae	Congo	Rainforest	Dimonika(04° 03′ 12″ S—12° 18′ 44″ E)	Afrotropical
	*Thoracotermes macrothorax*	Termitidae	Congo	Rainforest	Dimonika(04° 11′ 51″ S—12° 27′ 13″ E)	Afrotropical
Wood-feeding	*Microcerotermes parvus*	Termitidae	Congo	Rainforest	Dimonika(04° 08′ 47″ S—12° 15′ 36″ E)	Palaearctic, Neotropical, Afrotropical, Malagasy, Oriental, Papuan, Australian
	*Microcerotermes* spp.	Termitidae	Mexico	Grassland	La Mancha(19° 35′ 42″ S—96° 23′ 44″ E)	Palaearctic, Neotropical, Afrotropical, Malagasy, Oriental, Papuan, Australian
	*Nasutitermes nigriceps*	Termitidae	Mexico	Grassland	La Mancha(19° 35′ 54″ S—96° 22′ 34″ E)	Neotropical, Afrotropical, Malagasy, Oriental, Papuan, Australian
	*Nasutitermes voeltzkowi*	Termitidae	Mauritius Island	Wet forest	Macabé(20° 23′ 07″ S—57° 26′ 19″ E)	Neotropical, Afrotropical, Malagasy, Oriental, Papuan, Australian

### Experimental setup and gas sampling

Experiments were performed using sterile 60 mL serum vials (Wheaton Inc., Millville, USA). The setup comprised (i) live worker termites (0.5 g per vial); (ii) fragments of non-sterilized mound material (5 g) assembled as composite samples from different intact sections of the termite mound to reduce the effect of variations within these biogenic structures, (iii) a combination of live termites and termite non-sterilized mound material and (iv) empty vials.

The vials were sealed at ambient pressure with air-tight sterile butyl rubber septa and aluminum caps and were incubated in the dark at 27°C ± 2°C for 24 h as there is significant mortality for longer incubation times. There were three to five independent replicates for each termite species. Six milliliters of headspace gas were sampled immediately before incubation (T_0_) using a luer-lock syringe and transferred to 5.9 mL sterilized evacuated Exetainer^®^ vials (Labco Ltd., High Wycombe, England) for further analysis. This was replaced by 6 mL of ambient air to adjust the pressure. After 24-h incubation, the gas was sampled (T_24_) as described above and kept for analysis.

### N_2_O measurements and up-scaling

The N_2_O was measured using a gas chromatograph (CP-3800 VARIAN^®^ STAR; Agilent Technologies, Santa Clara, CA, USA), equipped with a ^63^Ni (15 mCi) electron capturing detector (ECD), as described by Majeed et al. [18]. The equipment was calibrated before the analyses with ambient air and commercial standards of helium and N_2_O (N48, Air Liquide, France) to give an accurate measurement of the N_2_O concentrations from 0 to 880 ppm. The detection limit of the GC system was 0.05 ppm. The chromatograms were processed using STAR_Workstation Version 6.0 (Agilent Technologies, Santa Clara, CA, USA) to determine the concentration of N_2_O in the sample. The results were scaled-up to estimate the total N_2_O production by termites in tropical forests by multiplying the gas emission rates by an estimate of the termite biomass density as described by Sanderson [20]. In this study, calculations were based on a termite biomass density of 337 kg ha^-2^ [20].

### Determining the ammonium and nitrate concentrations in the termite guts

About 100 mg of whole termite guts were crushed and homogenized in 5 mL of sterilized ultra-pure water. The samples were processed including shaking and centrifugation as described in ISO 14256–2:2005. The ammonium and nitrate in the supernatants of samples were quantified using a San^++^ Automated Wet Chemistry Analyzer (Skalar Analytical B.V., Netherlands). The ammonium and nitrate concentrations were determined by comparison with the standard curves obtained from samples of known concentrations and ranged from 0 to 4 mg L^-1^ for ammonium and 0 to 1 mg L^-1^ for nitrate. Three independent replicates were made for each treatment. Ammonium and nitrate contents were not determined for *Crenetermes albotarsalis* and *Nasutitermes nigriceps*.

### DNA extraction

Total genomic DNA was extracted from 200 mg whole termite-gut samples. Fast DNA^®^ SPIN Kit for Soil (MP Biomedical, Santa Ana, CA, USA) was used according to the manufacturer’s instructions. Extractions were made in triplicate for each treatment. The extracted DNA was verified by electrophoresis on a 1.5% (w/v) agarose gel which was then stained in a solution of ethidium bromide (0.5 mg L^-1^) for 15 min, rinsed for 20 min in milli-Q water and checked for the presence of DNA using an UV-transillumination system. The DNA was further quantified by PicoGreen^®^ (Molecular Probes, Inc., Eugene, Oregon, USA). The extracted DNA was stored at -20°C for further analysis.

### Quantitative PCR assay

The abundance of 16S rRNA gene and of the key functional marker genes involved in N cycling was estimated by quantitative PCR (qPCR), using a C1000^TM^ thermal cycler (CFX96^®^, BioRad, Hercules, California) as described by Majeed et al. [21]. The functional marker genes targeted included *amoA* encoding ammonia monooxygenase for ammonia-oxidizing archaea (AOA) and ammonia-oxidizing bacteria (AOB), and the *nirK*, *nirS* and *nosZ* denitrification genes, encoding copper-containing nitrite reductase, cytochrome *cd*_1_ nitrite reductase and nitrous oxide reductase, respectively. Primers targeting the 16S rRNA gene and the functional marker genes and thermal cycling parameters are given in S1 Table. The quantity of DNA was normalized before analysis and tested for PCR inhibition as described by Chapuis-Lardy et al. [22]. Standard curves were obtained from the serial dilutions of a known quantity of linearized pGEM®-T plasmids (Promega, Madison, WI, USA), carrying the targeted DNA sequences. The specificity of the amplified products was confirmed by melting curve analysis. The abundance of 16S rRNA gene and of the functional marker genes was not determined for *Thoracotermes macrothorax* and *Nasutitermes nigriceps*.

### Statistical analyses

Statistical analyses of N_2_O emissions by termites were performed using Statistica V 7.1 (StatSoft^®^ Inc., Tulsa, USA). Differences in levels of N_2_Oemission between termite species were based on the means of three or five replicates for each termite species. Measurements of the abundance of the genes targeted and the mineral N concentration in termite guts were based on eight or nine replicates. Prior to statistical comparison, the data were checked for normal distribution using Shapiro-Wilk test. The means were compared by One-Way Analysis of Variance (ANOVA) and then tested using Tukey’s HSD test with a level of significance of 0.05. The Pearson correlation coefficient was calculated to determine the degree of correlation between the N_2_O emissions and the abundance of the genes targeted and the mineral N concentration in the termite guts using R version 3.0.0. Results with a value of p< 0.05 were considered significant.

## Results

### Ammonium and nitrate concentrations in termite guts

Neither the ammonium nor the nitrate levels were related to termite feeding (Table 2). Ammonia concentrations ranged from 68.7 ± 5.7 to 899.6 ± 9.7 μmol (g dry wt.)^-1^ in the guts of the termite species studied and were higher than the nitrate concentrations. The highest ammonium concentrations were in the guts of a grass-feeding species, *Hodotermes mossambicus*, and a fungus-growing species, *Pseudacanthotermes militaris*. In each guild the concentrations of nitrate in the gut were below detection limit.

**Table 2:** Mineral N concentrations in gut of different termite species.

Feeding Group	Species	NitrateNO_3_^-^ μmol (g dry wt.)^-1^	AmmoniaNH_4_^+^ μmol (g dry wt.)^-1^
Fungus-growing	*Macrotermes muelleri*	0.004 ±0.002 ab	172.667 ±7.540 bc
	*Pseudacanthotermes militaris*	^_^	845.465 ±132.070 a
	*Pseudacanthotermes spiniger*	0.005 ±0.008 ab	259.115 ±7.643 bc
Grass-feeding	*Coarctotermes clepsydra*	0.003 ±0.002 ab	83.173 ±15.261 c
	*Hodotermes mossambicus*	^_^	899.635 ±9.663 a
	*Trinervitermes* spp.	0.045 ±0.011 a	703.296 ±33.661 a
Soil-feeding	*Capritermes capricornis*	^_^	310.670 ±18.394 bc
	*Cubitermes* spp.	0.033 ±0.008 ab	378.309 ±17.906 bc
	*Thoracotermes macrothorax*	0.004 ±0.009 ab	68.667 ±5.724 c
Wood-feeding	*Microcerotermes parvus*	^_^	251.653 ±23.609 bc
	*Microcerotermes* spp.	0.007 ±0.002 ab	93.108 ±6.807 c
	*Nasutitermes voeltzkowi*	^_^	158.884 ±16.268 bc

Values represent the means (± SE) of three independent replicates.

Values within a column with different lowercase letters indicate statistically significant differences (Tukey’s HSD test, p < 0.05).

(-) indicate that the concentration was below the detection limit.

Mineral N concentrations were not determined for *C. albotarsalis* and *N. nigriceps*

### Abundance of marker genes for ammonia-oxidizing and denitrifying processes

Functional marker genes for ammonia oxidation (*amoA*-AOA and *amoA*-AOB) and denitrification (*nirK*, *nirS* and *nosZ*) processes were successfully amplified from DNA extracts in all the termite guts tested and their copy numbers accounted for less than 10% of the total 16S rRNA gene (except for *Microcerotermes parvus*). There was no correlation between the abundance of these marker genes and the termite feeding guild to which the termite species belonged (Table 3). With the exception of *Crenetermes albotarsalis* and *M*.*parvus*, the copy numbers of the *amoA*-AOA marker gene were lower than the abundance of the *amoA*-AOB marker gene. For the nitrite reductase genes, the abundance of the *nirK* marker gene was higher than those of the *nirS* marker gene for all the species tested. The abundance of the *nosZ* marker gene, coding nitrous oxide reductase, was lower than the nitrite reductase marker genes (*nirK* and *nirS*) as indicated by the ratio *nosZ*/*nirK*+*nirS*. Overall, no relationship was found between the gene copy numbers of any of the functional genes tested and the feeding guild to which each species belonged.

**Table 3:** Copy numbers of 16S rRNA gene and functional marker genes for ammonia-oxidizing and denitrifying processes in gut of different termite species.

Feeding guild	Termite species	16S rRNA		Functional marker genes(copies ng^-1^ DNA)										Ratios	
				amoA-AOB		amoA-AOA		nirK		nirS		nosZ		amoA-AOA/amoA-AOB	nosZ/(nirK+nirS)
*Fungus-growing*	*M*. *muelleri*	7.07E+05 ^ab^	(1.98E+05)	1.41E+02 ^b^	(8.33E+01)	2.06E+02 ^b^	(1.94E+02)	3.85E+04 ^a^	(1.65E+04)	1.14E+03 ^b^	(3.19E+02)	1.24E+02 ^b^	(3.56E+01)	1.46	0.00
	*P*. *militaris*	3.33E+05 ^ab^	(2.57E+04)	1.08E+02 ^b^	(7.31E+00)	6.07E+00 ^b^	(2.70E+00)	2.98E+04 ^a^	(6.57E+03)	1.41E+03 ^b^	(3.09E+02)	4.59E+01 ^b^	(2.15E+00)	0.06	0.00
	*P*. *spiniger*	8.72E+04 ^b^	(3.35E+04)	5.81E+00 ^b^	(2.87E+00)	2.25E+00 ^b^	(1.54E+00)	3.19E+03 ^a^	(1.74E+03)	9.39E+02 ^b^	(3.72E+02)	1.06E+02 ^b^	(1.03E+02)	0.39	0.03
*Grass-feeding*	*C*. *clepsydra*	4.67E+05 ^ab^	(3.03E+05)	5.77E+01 ^b^	(1.23E+01)	2.29E+00 ^b^	(9.11E-01)	1.37E+04 ^a^	(1.09E+04)	4.93E+02 ^b^	(2.38E+02)	5.87E+01 ^b^	(3.20E+01)	0.04	0.00
	*H*. *mossambicus*	3.15E+05 ^ab^	(1.78E+05)	4.04E+01 ^b^	(1.62E+01)	4.88E+00 ^b^	(1.59E+00)	5.61E+03 ^a^	(3.59E+03)	2.10E+02 ^b^	(6.22E+01)	2.62E+02 ^b^	(2.21E+02)	0.12	0.05
	*Trinervitermes* spp.	5.91E+04 ^b^	(4.08E+04)	1.50E+00 ^b^	(6.77E-01)	3.56E-01 ^b^	(1.24E-02)	1.84E+02 ^a^	(1.01E+02)	3.19E+01 ^b^	(6.91E+00)	6.37E+00 ^b^	(3.84E+00)	0.24	0.03
*Soil-feeding*	*C*. *capricornis*	4.00E+05 ^ab^	(8.13E+04)	2.45E+02 ^b^	(1.22E+02)	7.60E+00 ^b^	(5.72E+00)	2.37E+04 ^a^	(4.94E+02)	2.75E+03 ^b^	(1.14E+03)	1.05E+03 ^a^	(4.05E+02)	0.03	0.04
	*C*. *albotarsalis*	7.99E+04 ^b^	(7.99E+04)	1.51E-01 ^b^	(8.47E-02)	5.97E-01 ^b^	(3.51E-01)	8.43E+02 ^a^	(8.41E+02)	1.30E+03 ^b^	(1.30E+03)	4.98E+02 ^ab^	(4.98E+02)	3.96	0.23
	*Cubitermes* spp.	9.78E+05 ^a^	(5.46E+05)	2.27E+01 ^b^	(1.15E+01)	2.29E+01 ^b^	(1.18E+01)	9.72E+03 ^a^	(4.67E+03)	1.33E+04 ^a^	(9.37E+03)	1.52E+02 ^b^	(9.66E+01)	1.01	0.01
*Wood-feeding*	*M*. *parvus*	1.81E+05 ^b^	(4.88E+04)	1.53E+02 ^b^	(5.96E-01)	1.73E+03 ^a^	(1.67E+02)	5.60E+04 ^a^	(5.52E+04)	5.70E+02 ^b^	(1.43E+01)	7.39E+02 ^a^	(3.65E+02)	11.37	0.01
	*Microcerotermes* spp.	7.75E+05 ^ab^	(5.44E+05)	6.17E+01 ^b^	(2.80E+01)	6.09E+00 ^b^	(5.79E-01)	4.45E+04 ^a^	(4.32E+04)	2.54E+03 ^b^	(1.56E+03)	1.95E+02 ^b^	(1.80E+02)	0.10	0.00
	*N*. *voeltzkowi*	7.22E+05 ^ab^	(2.64E+05)	1.84E+03 ^a^	(1.77E+03)	1.89E+01 ^b^	(3.78E+00)	2.98E+04 ^a^	(2.19E+03)	2.01E+03 ^b^	(8.01E+02)	3.03E+02 ^ab^	(1.80E+02)	0.01	0.01

Values represent the means (±SE) of three replicates.

Values within a column with different lowercase letters indicate statistically significant differences (Tukey’s HSD test, p < 0.05).

Gene copy numbers were not determined for *T. macrothorax* and *N. nigriceps*

### Levels of N_2_O production in different termite feeding guilds

**Fig 1:**
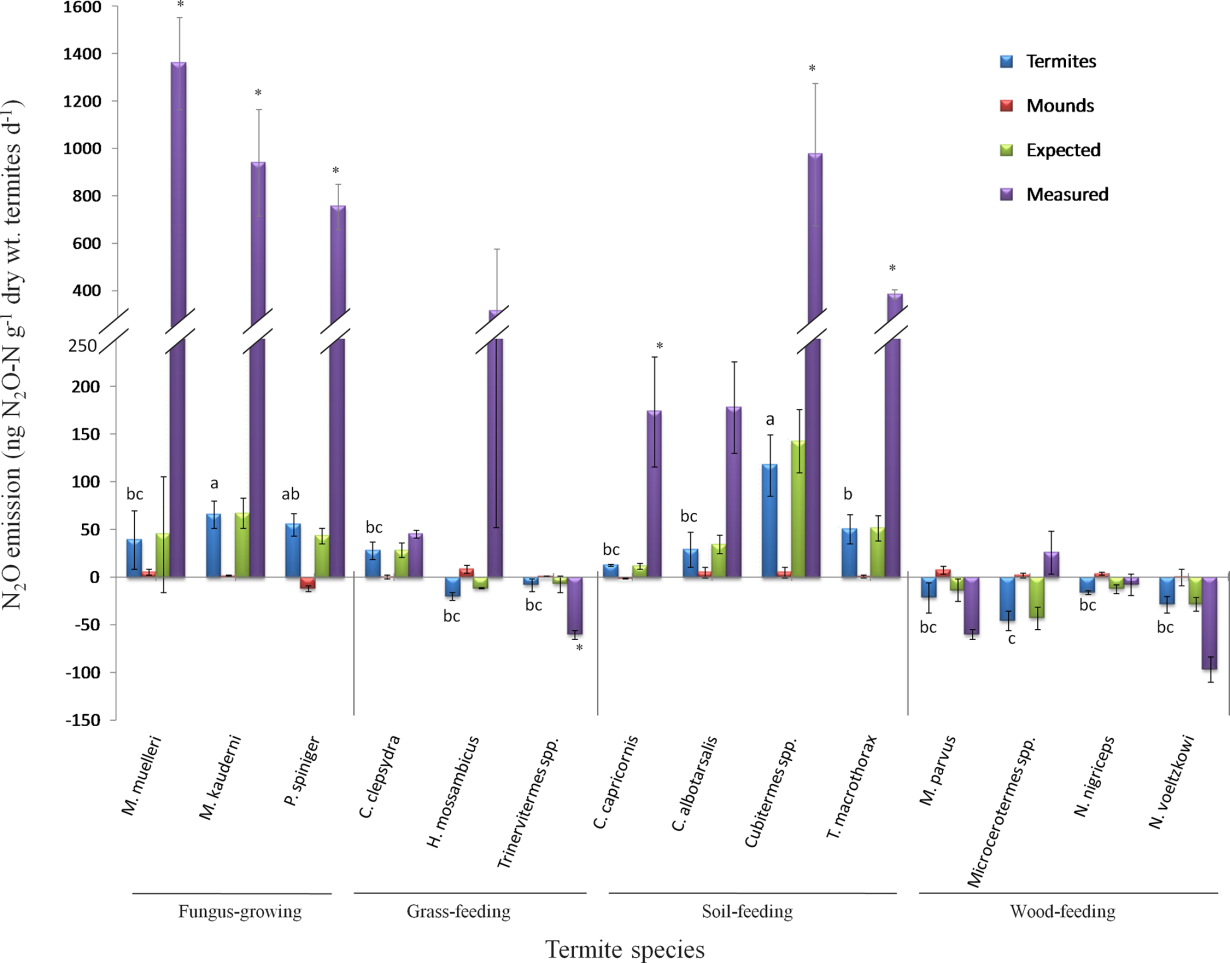
Levels of N_2_O production by termite species belonging to different feeding guilds. Bars represent means ± SE of three replicates. Expected: indicate N_2_O production based on the level of emissions measured separately for live termites and for termite mound. Measured: indicate the level of N_2_O production measured when termites were incubated together with their mound. Different letters mean that there was a statistically significant difference between termite species. * indicate statistically significant differences between level of N_2_O produced by termites alone and by termite incubated together with mound (Tukey’s HSD test, p < 0.05).

There was no termite mortality in any of the samples at the end of 24-h incubation. Differences in the mean N_2_O production between termite feeding groups (Fig 1) were statistically significant (*P* < 0.001). Fungus-growing and soil-feeding termites emit N_2_O. There were considerable differences in the levels of N_2_O production, ranging from 13.14 to 117.6 ng N_2_O-N d^-1^ (g dry wt.)^-1^ for soil-feeding species, with *Cubitermes* spp. having the highest production levels, and from 39.61 to 65.61 ng N_2_O-N d^-1^ (g dry wt.)^-1^ for fungus-growing species. Wood-feeding termites were net N_2_O consumers rather than N_2_O producers with a consumption ranging from 16.09 to 45.22 ng N_2_O-N d^-1^ (g dry wt.)^-1^.

This trend was also observed for two grass-feeding termite species (*H*. *mossambicus* and *Trinervitermes* spp.) but not for *Coarctotermes clepsydra* which is also a grass-feeder. Little N_2_O was produced from termite mounds incubated in the absence of live termites. When live termites were incubated with their mounds, N_2_O production increased by between 7and 13 fold for soil-feeders with the highest increase for *Cubitermes* spp. and between 14 and 34 fold for fungus-growers with highest increase for *M*. *muelleri* (Fig 1). This increase is higher than the sum of N_2_O produced separately by live termites and by termite mound material (expected production). The differences were statistically significant for fungus-growing termites and for soil-feeding termites except for *C*. *albotarsalis*. For the wood-feeding species, *M*. *parvus* and *Nasutitermes voeltzkowi*, an increase of about 3-fold in the level of N_2_O consumption was obtained in the presence of termite mound material. However, this was not observed for *N*. *nigriceps* and *Microcerotermes* spp. Overall, the levels of N_2_O production were not correlated with the termite gut mineral N concentration or with the copy numbers of ammonia-oxidizing and denitrifying marker genes in the gut.

## Discussion

### Are the levels of N_2_O production affected by the termite feeding guild?

The pattern of N_2_O production shows that the levels of N_2_O emissions depend on the termite feeding guild. Termites feeding on soil and fungi produced N_2_O. In general, while termites feeding on soil and fungi were a source of N_2_O, wood-feeding and grass-feeding termites were a sink for N_2_O. Further studies using more species are needed to assess the consumption of N_2_O in wood-feeding and grass-feeding termites. Overall, our results are consistent with the initial hypothesis that the termite feeding guild is a determinant of N_2_O production although the average emission levels presented here may not accurately reflect the initial rates at the start of incubation. Cumulative gas production was measured after 24-h incubation in vials (rather than measuring a time series) to ensure that the levels of N_2_O emissions were above the analytical detection limits. This approach may have underestimated the actual rates. However, the levels of N_2_O production were not limited by the experimental design and the duration of the experiment was less than the time taken for food to pass through the gut of soil-feeding termites. For the wood-feeding termite species studied, the levels of N_2_O remaining in the vial headspace after the incubation period accounted for between 10–30% of the total initial quantities. This indicates that, for these termite species and their gut microorganisms, the low N_2_O concentration in the ambient air did not affect N_2_O uptake rates through substrate limitation. The zero mortality of termites in all treatments might indicate that not all the oxygen in the vial headspace was depleted by termite respiration.

These results confirm previous findings on N_2_O production by soil-feeders and N_2_O consumption by wood-feeders [16, 18]. They also show that some fungus-growing termite species are also a source of N_2_O and some grass-feeding species may consume atmospheric N_2_Oin the same way as wood-feeders. The average N_2_O production level for *Cubitermes* spp., which is considered to be a true soil-feeding termite, was much higher than for the other termites studied but was about 4-fold lower than reported for *Cubitermes ugandensis* [16].

There was no correlation between the N_2_O emission rates and the concentrations of ammonia and nitrate measured in the termite guts. This confirms that pool sizes of metabolites are not good indicators of the emission rate because the mineral-N pool does not adequately reflect N availability [23, 24].

To simulate the termite N_2_O metabolism under field conditions, live termites were incubated in the presence of their mound material. The higher increase in levels of N_2_O production under these conditions might be explained by the use of mound material as a food by termites during incubation. Under these conditions, the production of N_2_O might have remained constant whereas N_2_O production may have decreased during incubation without mound material as a result of starvation. The increases in N_2_O production in this study are greater than the increases reported previously in soil microcosms for *C*. *ugandensis* and *Ophiotermes* spp. [16]. This discrepancy might be explained by the differences in the quantities of mineral N ingested by the termites. Mounds of soil-feeding termites have much higher concentrations of mineral N (ammonia and nitrate) than soils [13, 25]. Incubating termites with termite mound fragments results in continuous ingestion of considerable amounts of mineral N and the transformation of the ingested nitrate may increase N_2_O emissions. It has been established that ingested nitrate is denitrified to N_2_ or reduced to ammonia in the guts of soil-feeding termites [15, 16]. This study did not measure N_2_ emissions as it did not set out to determine denitrification pathways in termite guts. Further studies are needed to assess whether or not the denitrification process is complete for various termite species belonging to a particular feeding guild.

### Are mineral N and the abundance of marker genes for ammonia-oxidizing and denitrifying bacteria in the guts related to termite diet?

There is little information on mineral-N in the guts of termites belonging to different feeding guilds. However, it has been well documented that there are high ammonia concentrations in the posterior hindgut of soil-feeding species [13, 15, 16]. Our results suggest that high ammonia concentrations in the termite gut are not exclusive to soil-feeding termites as the guts of species belonging to other feeding guilds, such as wood-feeding, grass-feeding and fungus-growing termites, also had high ammonia concentrations. Nitrate concentrations in *Cubitermes* species were considerably lower than the ammonia concentrations as reported elsewhere [15, 16]. This supports the prevalence of nitrate ammonification processes in the gut of soil-feeding termites [15, 16]. In this study, the ammonia concentrations in the gut of soil-feeding termite species were in the same range as the overall concentrations in the whole gut of *Cubitermes* species [15]. Higher ammonia concentrations found in soil-feeding and fungus-growing termite species have been explained by the fact that (i) a large proportion of the food of soil-feeding termites is derived from peptides and other nitrogenous humus components of soils [26], (ii) the existence of a nitrate ammonification process in the termite gut [15, 16], (iii) the acquisition of nitrogen by fungus-growing termites from their symbionts, the *Termitomyces*, a rich source of nitrogen [27].

Given the differences in the levels of N_2_O production between the termites species studied, the relationship between the feeding guild and the copy numbers of marker genes of ammonia-oxidizing (*amoA*-AOB; *amoA*-AOA) and denitrifying bacteria (*nirK*, *nirS* and *nosZ*) in termite guts was investigated. Unlike *nifH* genes involved in the fixation of atmospheric N_2_ [28–31], very little is known about marker genes for ammonia-oxidizing and denitrifying processes in termite guts. This study records, for the first time, the presence of the key functional genes involved in these processes in the guts of termite species belonging to a number of different feeding guilds. The abundance of these marker genes did not depend on the feeding guild. This was unexpected since diet can be a major determinant of the bacterial community structure in termite guts [19]. Measuring the abundance of marker genes of ammonia-oxidizers and denitrifiers in the intestinal gut as a whole (as in this study) does not take account of the differences in the composition of the microbial community in each gut compartment which depends on the prevailing physical and chemical conditions [32]. Further studies are needed to determine the expression levels of the marker genes for ammonia-oxidizing and denitrifying bacteria in the various gut compartments. Particular attention should be paid to the genes involved in the dissimilatory nitrate reduction to ammonium since this is the prevailing pathway by which N_2_O is also produced in termite guts of soil-feeding species [16].

### N_2_O emissions by termites and ecological outcomes

Termites have a high biomass in many tropical biomes and have been recognized as ecosystem engineers mediating various biological soil functions including biogeochemical processes [5]. There is still little information on the contribution of termites to the N_2_O budget in tropical forests. Soil-feeding termites are more abundant and have greater biomass in tropical forests and humid African forests, whereas Macrotermitinae (fungus-growing termites) are dominant in arid and semi-arid savannas [33]. Taking the highest average levels of N_2_O production (117.6 ng N-N_2_O d^-1^ (g termite dry wt.)^-1^) recorded in this study for the *Cubitermes* spp., which is a true soil-feeding termite species, and an estimate of the biomass of soil-feeding termite species in tropical forests (337 kg ha^-1^; [20]), the production of N_2_O by termites belonging to the soil-feeding guild was estimated at 0.39 kg N-N_2_O ha^-1^ y^-1^.

This is about 4-fold lower than calculations based on the levels of N_2_O reported previously for *C*. *ugandensis* [16]. However, the contribution of termites to the atmospheric N_2_O budget is probably higher since the presence of mound material increases N_2_O production, as shown in this study. A previous study identified the mounds of soil-feeding termites as hot spots of N_2_O emission in the African savannah [17]. This is confirmed by the positive interaction between termite mound material and live termites for N_2_O production observed in this study. These findings suggest that scaling up calculations for termite-mediated N_2_O emissions to the atmosphere should take account of the field conditions.

The N_2_O uptake by wood-feeding termites is interesting from an ecological point of view. Unfortunately, it was difficult to scale up calculations of N_2_O consumption by wood-feeding termites as there is a lack of data on their abundance and biomass in the different biomes. It has been established that species in the wood-feeding guild tend to replace soil-feeding termites in response to forest disturbances [4, 34]. Therefore, wood-feeding termites might be important players in scenarios for mitigating N_2_O emissions into the atmosphere.

The most notable conclusion from this study suggests that the composition of termite feeding guild assemblages in ecosystems should be taken into account when scaling up the contribution of termites to the global budget of N_2_O emissions. Land-use change might lead to the propagation of one termite feeding guild at the expense of another, and could lead to increased or reduced N_2_O emissions.

## Supporting Information

S1 Table Primers used in this study for real-time PCR quantification of 16S rRNA genes and functional marker genes for ammonia-oxidizing and denitrification in gut of different termite species. (DOCX)
