# Supplementary material for: Nitrous Oxide (N2O) Emissions by Termites: Does the Feeding Guild Matter?
Source: PLoS One. 2015 Dec 10;10(12):e0144340. doi: 10.1371/journal.pone.0144340 (PMC4675541; doi:10.1371/journal.pone.0144340)
Supplement: S1 Table — (DOCX) [file pone.0144340.s001.docx]

| Target gene | Fragment length | Primers | Gene positions | Thermal cycling conditions | Sequence (5'-3') | Reference |
| --- | --- | --- | --- | --- | --- | --- |
| *16S rRNA* | *174 bp* | *341F* | 341–357 | 1 cycle 95°C -15 min; followed by 35 cycles 95°C - 15 sec, 60°C -30 sec, 72°C - 30 sec, 80°C - 30 sec | CCTACGGGAGGCAGCAG | Lopez-Gutiérrez *et al*. 2004 |
|  |  | *515R* | 491-515 |  | ATTCCGCGGCTGGCA |  |
| *amoA-AOA* | nd | CrenamoA23F | 7-631 | 1 cycle 95°C -15 min; followed by 40 cycles 95°C - 15 sec, 55°C -30 sec, 72°C - 30 sec, 80°C - 30 sec | ATGGTCTGGCTWAGACG | Tourna *et al*. 2008 |
|  |  | CrenamoA616R |  |  | GCCATCCATCTGTATGTCCA |  |
| *amoA-AOB* | *491 bp* | AmoA1F | 332-349 | 1 cycle 95°C -15 min; followed by 40 cycles 95°C - 15 sec, 55°C -30 sec, 72°C - 30 sec, 80°C - 30 sec | GGGGTTTCTACTGGTGGT | Rotthauwe *et al*. 1997 |
|  |  | AmoA2R | 802-822 |  | CCCCTCKGSAAAGCCTTCTTC |  |
| *nirK* | *165 bp* | nirK876 |  | 1 cycle 95°C -15 min followed by 6 cycles 95°C - 15 sec, 63°C - 1°C / cycle - 30 sec, 72°C - 30 sec, 80°C - 30sec followed by 40 cycles 95°C - 15 sec, 58°C - 30 sec, 72°C - 30 sec, 80°C - 30 sec; melt curve 80°C - 95 °C | ATYGGCGGVAYGGCGA | Braker *et al*. 1998; Henry *et al*. 2004 |
|  |  | nirK1040 | 1023-1040 |  | GCCTCGATCAGRTTRTGGTT |  |
| *nirS* | *890 bp* | cd3aF | 916-935 | 1 cycle 95°C -15 min followed by 6 cycles 95°C - 15 sec, 63°C - 1°C / cycle - 30 sec, 72°C - 30 sec, 80°C - 30sec followed by 40 cycles 95°C - 15 sec, 58°C - 30 sec, 72°C - 30 sec, 80°C - 30 sec; melt curve 80°C - 95 °C | GTSAACGYSAAGGARACSGG | Throbäck *et al*. 2004 |
|  |  | R3cd | 1322-1341 |  | GASTTCGGRTGSGTCTTSAYGAA |  |
| *nosZ* | *267 bp* | nosZ2F | 1617-1640 | 1 cycle 95°C-15min followed by 6 cycles 95°C-15sec, 63°C-1°C/cycle-30sec, 72°C-30sec, 80°C-30sec, followed by 40 cycles 95°C-15sec, 58°C-30sec, 72°C-30sec, 80°C-30sec; melt curve 80°C-95°C | CGCRACGGCAASAAGGTSMSSGT | Henry *et al*. 2006 |
|  |  | nosZ2R | 1864-1884 |  | CAKRTGCAKSGCRTGGCAGAA |  |

**Table S1** Primers used in this study for real-time PCR quantification of 16S rRNA genes and functional marker genes for ammonia-oxidizing and denitrification in gut of different termite species

Ambiguity codes: R = A/G; N = A/G/C/T; H = T/C/A; Y = C/T; S = C/G; K=G/T; B=G/C/T;
